# Differences in learning rates for item and associative memories between amnestic mild cognitive impairment and healthy controls

**DOI:** 10.1186/1744-9081-9-29

**Published:** 2013-07-25

**Authors:** Pengyun Wang, Juan Li, Huijie Li, Shouzi Zhang

**Affiliations:** 1 Center on Aging Psychology, Key Laboratory of Mental Health, Institute of Psychology, Chinese Academy of Sciences, 16 Lincui Road, Beijing, 100101, China; 2 Graduate School, Chinese Academy of Sciences, Beijing, China; 3 Beijing Geriatric Hospital, Beijing, China

**Keywords:** Mild cognitive impairment, Associative memory, Item memory, Learning rate

## Abstract

**Background:**

It has been established that the overall performance of associative memory was disproportionately impaired in contrast to item memory in aMCI (Amnestic mild cognitive impairment) patients, but little is known about the specific aspects of the memory process that show differences between aMCI and healthy controls. By comparing an item-item associative learning test with an individual item learning test, the present study investigated whether the rate of learning was slower in associative memory than in item memory in aMCI. Furthermore, we examined whether deficits in intertrial acquisition and consolidation contributed to the potential disproportionate impairments in the learning rate of associative memory for aMCI patients. In addition, we further explored whether the aMCI-discriminative power of the associative memory test increases more than that of the item memory test when the number of learning-test trials increases.

**Methods:**

A group of 40 aMCI patients and 40 matched control participants were administered a standardized item memory test (Auditory Verbal Learning Test, AVLT) and a standardized associative memory test (Paired Associative Learning Test, PALT), as well as other neuropsychological tests and clinical assessments.

**Results:**

The results indicated that the learning rate deficits in aMCI patients were more obvious for associative memory than for item memory and that the deficits resulted from impairments in both intertrial acquisition and consolidation. In addition, the receiver operating characteristic curve and logistical regression analysis revealed that the discriminative power of the associative memory test for aMCI was larger than that of the item memory test, especially with more than one learning-test trials.

**Conclusions:**

Due to more deficits in learning rate of associative memory than that of item memory, the discriminative power for aMCI tended to be larger in associative memory than in item memory when the number of learning-test trials increased. It is suggested that associative memory tests with multiple trials may be particularly useful for early detection of aMCI.

## Background

Mild cognitive impairment (MCI) is a state with mildly impaired cognitive functions but intact ability to perform basic daily activities. It is generally considered as a transitional stage between normal aging and a diagnosis of probable clinical dementia [1]. Amnestic MCI (aMCI) is a subtype of MCI in which patients show early episodic memory impairment but do not fulfill the criteria for dementia [2]. A meta-analysis indicated that the annual conversion rate from MCI to dementia is approximately 5–10% [3], which is obviously higher than the incidence rates form normal elderly to dementia (1–2% per year) [1]. It is thus important to investigate the nature of episodic memory deficits in people with MCI/aMCI and to identify them at a very early stage.

Episodic memory tests with multiple learning-test trials are more sensitive than those with single learning–test trial for the diagnosis of MCI [4-9]. A multi-trial learning test involves complex memory processes, such as memory span, learning rate, and the ability to acquire and consolidate information, which may take different impaired patterns in AD and in aMCI patients [10-12]. Thus, some important information might have been overlooked in the usual summary statistics by only relying on the comparison of total memory performance, and thus the memory impairment in multi-trial learning tests of aMCI patients needs be investigated in more detail.

Item memory and associative memory involve distinct processes and underlie different neural structures [13]. Item memory involves remembering individual items, such as a word or an object, while associative memory requires remembering of the relationship between items, such as pairs of words [14]. It has been well established that associative memory has been found disproportionately impaired in contrast to item memory in aMCI population [14,15] in terms of the overall performances, but little is known about the specific aspects of the memory process (such as learning rate) that show differences between aMCI and healthy controls.

Therefore, in the present study, we attempt to compare the performance on a multi-trial associative learning test (Paired Associative Learning Test, PALT) [16] with performance on a multi-trial item learning test (Auditory Verbal Learning Test WHO/UCLA version, AVLT) [17,18] to examine the disproportionate associative memory deficits in aMCI in depth in terms of learning rate, intertrial acquisition and consolidation.

### Rate of learning

Lezak (2004) suggested that memory assessors distinguish between memory span and learning increment [19]. Memory span is a measure of the capacity to learn information in a single trial. Learning increment reflects rate of learning, that is, the ability to integrate information from trial to trial. This can be deduced from the slope of the learning curve in multiple learning trials. Learning rate has been found to be sensitive to age-related memory deficits and even to stimuli characteristics (word frequency) in older adults [20]. People with cerebral lesions in different brain locations also showed different learning rates [21]. There is accumulating evidence that AD patients have a shallower slope of the learning curve than controls for learning tasks that use repeated items [12,22]. As an early stage of AD, experimental evidence has also revealed that people with MCI exhibit a deceleration of learning in tests that use word lists [12,23].

The rate of learning in associative memory has also been found to be impaired in people with aMCI compared to normal older adults [14,24]. But little is known about whether the learning rate in associative memory decelerates more than in item memory. To our knowledge, only Troyer et al. (2008) [14] examined this issue, but they did not find any difference in the impairment of learning rate between item and associative memory. However, the memory test used in that study (Brief Visuospatial Memory Test—Revised) [25] consisted of only six items and six associations in each trial and the mean score of the control group on associative memory was 5.1 in Trial 2 and 5.5 in Trial 3. Thus, there might have been a ceiling effect, resulting in a non-significant interaction between learning trial, group, and memory type. Therefore, the first aim in the present study was to confirm if learning rate for associative memory in individuals with aMCI was more impaired than for item memory.

### Intertrial acquisition and consolidation

To improve performance in learning tasks involving multiple trials, an individual must carry out two processes. One is acquiring items that have not yet been learned well enough to be retrieved. The other is consolidating items that have already been acquired [26]. Hence, seemingly identical learning curves may result from distinct contributions of acquisition and consolidation between trials.

Thus, a meaningful method to investigate learning rate is to consider intertrial performance, i.e., the examination of the items that are gained and lost on multiple repeated trials [12,20,22]. Trial-by-trial performance can be analyzed according to gained access (GA; the proportion of items recalled on trial *n* + 1 out of those that were not recalled on trial *n*) and lost access (LA; the proportion of items not recalled on trial *n* + 1 out of those that had been recalled on trial *n*) [20,26]. GA mainly reflects intertrial acquisition and can be presumed to be a function of the degree to which a representation of an item in memory is strengthened during a particular study trial. On the other hand, LA mainly reflects intertrial consolidation deficits that lead to rapid intertrial forgetting and can be conceptualized as the proportion of items that do not possess sufficient strength to be recalled consistently [22]. We would acknowledge here that in addition to acquisition and consolidation, retrieval may also contribute to both GA and LA e.g. [12,20] and two variables of GA and LA may not be mutually exclusive. Nevertheless, following Almond et al. (2013) [20], we shall accept the general consensus that acquisition and consolidation account for the majority of GA and LA, respectively.

This approach is particularly pertinent to our understanding of the feature of learning rate in aMCI patients. Moulin et al. (2004), using a 10-word multiple-trial learning task, found that both AD and MCI patients showed lower GA and greater LA of items between trials relative to normal controls [12]. This suggested that both failure of intertrial acquisition and consolidation contribute to the milder learning curve in item memory in MCI and AD patients. However, little is known about the manner by which acquisition or consolidation deficits may combine to limit the performance of people with aMCI across individual study-test trials in associative memory. Thus, the second purpose of the current study is to extend this deconstructive approach to PALT in order to investigate whether the deficit in learning rate for associative memory in aMCI patients is due to impairment in both intertrial acquisition and consolidation, similar to that in item memory.

### Discriminative power for aMCI

Multiple-trial learning test tasks seemed more powerful than the tasks with single learning-test trial for MCI diagnosis [4-6]. This may be because group differences accumulate with multiple trials. Thus, if a difference in learning rate between associative memory and item memory existed, then the discriminative power of the associative memory test for aMCI should increase more than that of the item memory test with increasing learning trials. Therefore, the third purpose of the current study is to investigate whether aMCI discriminative power of associative memory tended to be larger than that of item memory when the number of learning-test trials increased.

## Method

### Participants

#### Recruitment and classification of participants

All participants were from a community- based MCI screening project [27]. They completed a battery of neuropsychological tests, a clinical assessment, and neuroimaging examinations when applicable. The clinical assessment included a survey of participants’ medical history, a basic physical exam, as well as the Neuropsychiatric Inventory (NPI), Activities of Daily Life (ADL with 14 items), the Global Deterioration Scale (GDS), Clinical Dementia Rating (CDR), Hachinski Ischemic Score (HIS), and Structured Clinical Interview for DSM Disorders (SCID, depression and anxiety parts only). Research assistants with psychological background administered the neuropsychological battery, and experienced psychiatrists were responsible for the clinical assessment. All the research assistants and clinicians were intensively trained with a high inter-rater reliability (above 90%). The screening process was standardized with a comprehensive Case Report Form (CRF) recorded for each participant.

The diagnostic criteria for aMCI followed the criteria suggested by Petersen et al. (2001) [2]: (1) subjective complaints of memory loss, preferably corroborated by an informant; (2) evidence of objective memory impairment confirmed by one standard deviation (SD) below the expected levels for age and education on the visual recognition test (a subtest of the World Health Organization Neuropsychological Battery of Cognitive Assessment Instruments for the Elderly, WHO-BCAI) [18,28]; (3) normal general cognitive functioning confirmed by MMSE scores (MMSE score ≥ 24 for those who had received more than 7 years of education and MMSE score ≥ 20 for those who had received less than 7 years of education) [21]; (4) a global CDR score of 0.5 with a memory score of 0.5 or 1; (5) level 2 or level 3 on the GDS; (6) intact activities of daily life (ADL ≤ 18); (7) HIS < 4; and (8) an absence of dementia. For the memory cut-off scores, we adopted a more liberal criterion of one SD below the age- and education-corrected norm following Troyer and Murphy (2007) [29], because previous studies have suggested that the traditional 1.5 SD cut-off would reduce the possibility of detecting early memory impairment [30].

The inclusion criteria for the NC group were as follows: (1) normal general cognitive function; (2) normal objective memory; (3) a global CDR score of 0; (4) intact activities of daily life (ADL ≤ 18); and (5) HIS < 4.

The exclusion criteria for both groups were as follows: (a) significant visual and/or auditory impairment; (b) current diagnosis of, or history of significant medical, neurological, or psychiatric illness (such as depression and anxiety), and (c) history of alcohol or substance abuse. In addition, the participants with possible floor or ceiling effects were also excluded, as their recall scores were zero or full for some trials, and GA cannot be calculated for the trial with full marks, and LA cannot be calculated for the trial with zero marks.

The AVLT and PALT were not used to diagnose aMCI in the present study. This study was approved by the ethics committee of the Institute of Psychology, Chinese Academy of Sciences. Written informed consent was obtained from each participant.

#### Demographic characteristics and group differences

Forty older adults diagnosed with aMCI (14 men, 26 women) and 40 healthy older adults (normal control; NC; 17 men, 23 women) were included in the present study. Parts of the demographic and neuropsychological characteristics of the aMCI and NC groups are presented in Table 1. All aMCI participants were closely matched with healthy older adult participants in terms of age, gender, and education. Individuals with aMCI scored significantly lower on the Mini-Mental State Examination (MMSE) although all of their scores were in the normal range. The aMCI patients scored significantly lower than the NC group in working memory (Digit Span Forward and Backward Subtest, Chinese version of the Wechsler Memory Scale-Revised) [31], visual spatial ability (Chinese version of Block Design Subtest of the Wechsler Intelligence Scale Revised) [32], and language ability through a verbal fluency test [33], compared to the NC group. There were no significant group differences in semantic memory, which was determined through a category fluency test [33].

**Table 1:** Demographic data and neuropsychological test data by group

**Variable or test**	**NC (n = 40)**		**aMCI (n = 40)**		***t***/**χ**^**2**^	***p***	**Cohen**’**s *d***
	**Mean**	**SD**	**Mean**	**SD**			
Age (in years)	69.95	6.63	68.78	6.04	0.83	.410	0.19
Education (in years)	8.70	3.15	8.53	3.80	0.22	.823	0.05
Gender	23 F/17 M		26 F/14 M		0.47	.491	-
MMSE	27.95	1.84	25.73	2.69	4.32	<.001	0.98
Visual recognition	14.20	1.16	10.55	1.23	13.60	<.001	3.09
Digit span (forward)	9.98	1.83	8.80	2.84	2.20	.031	0.50
Digit span (backward)	5.63	2.08	4.53	1.74	2.56	.012	0.58
Verbal fluency (fa)	7.40	3.32	5.90	3.15	2.07	.042	0.47
Category fluency (vegetables)	15.48	4.44	15.05	3.82	0.46	.647	0.11
Block design	27.85	8.10	22.58	7.89	2.95	.004	0.67

*Notes*. F = female, M = male. Score of visual recognition = corrected recognition - incorrect recognition + 8.

According to the definition of aMCI subtypes by Petersen et al. (2004) [1], if memory is the only impaired domain, then individuals are classified into the aMCI-single domain. If other domains such as language, attention/executive function, or visuospatial skills are also impaired in addition to memory, then individuals are classified into the aMCI-multiple domain. Unfortunately, there are no large Chinese elderly sample based norms available for the cognitive tests used in current study. Thus, we were unable to classify the aMCI patients in our study as single- or multi- domain. However, the lower performance of the aMCI group on the various neuropsychological tests suggests that a large portion of the aMCI patients in the present study may be classified as aMCI-multiple domain.

### Measures and procedure

#### *AVLT*

The administration of the AVLT (WHO-UCLA version) involves study and test trials of two lists of 15 concrete nouns (one critical list and one distractor list). Stimulus words were read aloud at a rate of approximately one word per second. Participants were instructed to remember as many words as possible and to recall them in any order. Words on the critical list were presented in the same order for five study-test trials. After the fifth test trial of the critical list, participants performed 20 min of non-memory-related activities, after which they were again asked to recall the words from the critical list. Subsequently, the distractor list was presented for one study-test trial. Immediately following this study-test trial of the distractor list, participants were asked to recall all the words from the critical list. The timing of the presentation of the distractor list was adjusted after the fifth study-test trial in the initial WHO/UCLA version [17] in order to obtain a delayed recall performance unaffected by the distractors. Only the first three study-test trials were analyzed such that the number of trials for the PALT and AVLT were equal.

#### *PALT*

The PALT consists of 12 word pairs, with six easy pairs (the words within each pair have semantic relationship) and six difficult pairs (the words within each pair do not have semantic relationship). Each word is made up of two Chinese characters. In the study phase, the word pairs were read at a rate of approximately one word pair per second, with the interval between two pairs being 2 s. After the study list was presented, participants were required to recall the second word of each pair within 5 s once the experimenter had read the first word. This procedure was then repeated twice, with the words in a different order [16].

There was no significant difference for the to-be-remembered items between the two tests in terms of word frequency or age of acquisition (Additional file 1).

### Statistical analyses

In order to compare the scores on the PALT and AVLT, all the scores in both tests were standardized to represent the proportion of correct items. Statistical analyses were carried out with the Statistical Package for Social Sciences version 20.0 (SPSS Inc., Chicago, IL). A t-test and a chi-square test (alpha level = .05) were performed to examine group differences in demographic variables and other neurocognitive function measures. The effect sizes of the group comparisons were calculated in terms of Cohen’s d [34].

Mixed-design analyses of variance (ANOVAs; alpha level = .05) were conducted to analyze the learning curve, GA, and LA of both tests. The between-subjects factor was group (NC vs. aMCI) for all ANOVAs. For the analysis of the learning curve of the PALT, the within-subjects factors were difficulty level (easy vs. hard) and trial (three trials). Trial (three trials) was the only within-subjects factor in the analysis of the AVLT learning curve. Similarly, the within-subjects factor for the GA and LA analyses was trial (three trials for GA and two trials for LA in both two tests). The Bonferroni correction was applied for multiple comparisons among three trials for PALT/AVLT analysis, and among three gains/trials for GA analysis. The degrees of freedom for the within-subjects comparisons were corrected for deviance from sphericity (Greenhouse-Geisser).

The Pearson product–moment correlation (alpha level = .05) was conducted to test whether the GA and LA were correlated, and whether memory span (Trial 1) and learning rate (Trial 3 minus Trial 1) were correlated in both tests. Both the AUC from ROC curve analysis and the percentage of accurate classification from the logistic regression analysis were employed to compare the discriminative power of the PALT and AVLT for detecting aMCI separately.

All the ANOVA analyses for learning rate, GA, and LA were conducted again with education, age, digit span forward, digit span backward, verbal fluency, and block design scores as covariates. The results did not differ from those of the initial analyses. Since there were slightly more women than men in the sample, we carried out all the ANOVAs again with gender as another between-subject factor. The results showed no significant main effect of gender or any interactions with gender, indicating that gender did not influence the present results. For simplification, only the original results are reported in the following section.

## Results

### Rate of learning

#### *AVLT*

**Figure 1:**
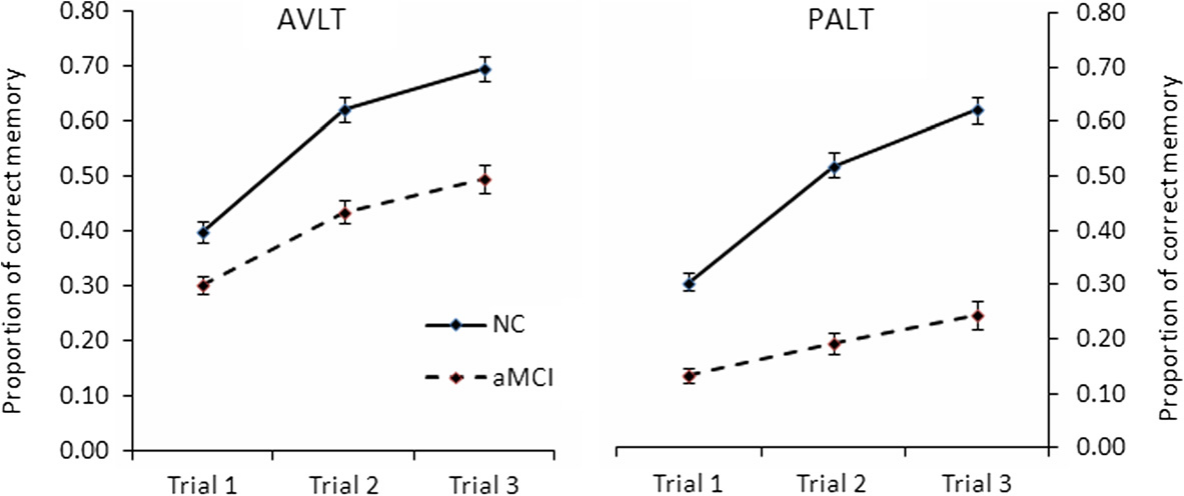
**Learning curves on the AVLT and PALT.** Bars depict standard error of the means (SEM)

The analysis of trials 1 through 3 revealed that the aMCI group generally performed worse than NC, *F* (1, 78) = 30.68, *p* < .001, η^2^ _*p*_ = 0.28. A significant main effect of trial was found, *F* (2, 77) = 101.02, *p* < .001, η^2^ _*p*_ = 0.72. A pairwise comparison indicated that trial-by-trial performance increased significantly (all *p*s < .001). Moreover, there was a significant interaction between group and trial, *F* (2, 77) = 4.85, *p* = .010, η^2^ _*p*_ = 0.11. Subsequent simple effect analyses revealed that AVLT scores increased significantly over the three trials for both groups, all *p*s < .05. As observed in Figure 1-AVLT and the examination of the scores and effect sizes, this interaction is due to a shallower slope of the learning curve from trials 1–3 for aMCI as compared to NC, resulting in larger group differences on later learning trials (see Figure 1-AVLT).

#### PALT

The results showed that NC performed better than aMCI across difficulty levels and trials, *F* (1, 78) = 130.05, *p* < .001, η^2^ _*p*_ = 0.63. Scores on difficult word pairs were lower than those on easy word pairs, *F* (1, 78) = 425.77, *p* < .001, η^2^ _*p*_ = 0.85. There was also a significant main effect of trial, *F* (2, 77) = 101.84, *p* < .001, η^2^ _*p*_ = 0.73. Pairwise comparison analysis indicated that memory scores increased significantly over the three learning trials (all *p*s < .001). The interaction between trial and group was significant, *F* (2, 77) = 26.97, *p* < .001, η^2^ _*p*_ = 0.41. Subsequent simple effect revealed that the interaction reflected significant trial differences on both groups, with the differences greater in NC, *F* (2, 156) = 138.17, *p* < .001, than in aMCI, *F* (2, 156) = 15.91, *p* < .001, suggesting that learning rate was significantly slower in aMCI than in NC, which resulted in larger group differences in later learning trials. The interaction between group, trial, and difficulty level was not significant, Greenhouse–Geisser adjusted *F* (1.77, 138.02) < 1, indicating that the pattern was the same for both difficulty levels. Thus, easy and difficult word pairs were collapsed together to compare the learning curves between PALT and AVLT (learning curves with average scores for easy and difficult word pairs are presented in Figure 1-PALT).

#### Differences in learning rate between AVLT and PALT

**Figure 2:**
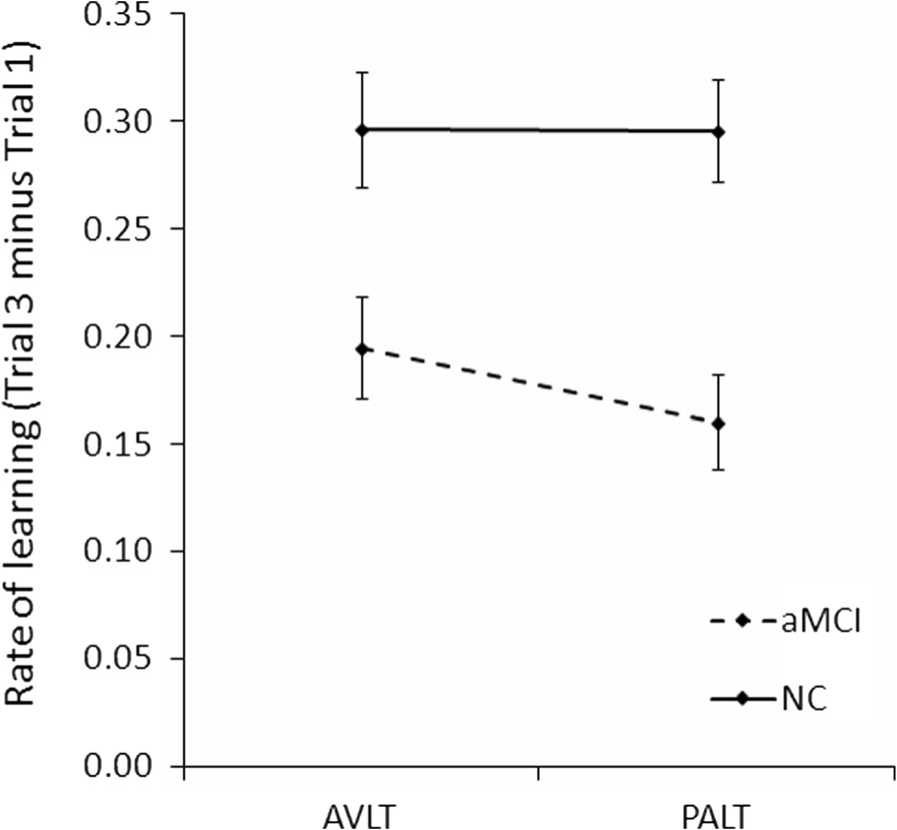
**Comparison of the learning rates on the AVLT and PALT.** Bars depict standard error of the means (SEM).

In order to compare differences in learning rate between AVLT and PALT, we analyzed memory increment between the first and third trial in both tests. A mixed-design ANOVA was conducted between group (between-subjects factor; aMCI and NC), test type (within-subjects factor; PALT and AVLT), and trial (within-subjects factor; trials 1 and 3). The results revealed a significant 3-way interaction, *F* (1, 78) = 6.14, *p* = .015, η^2^ _*p*_ = 0.07. Subsequent simple effect analysis and examination of the scores and effect sizes indicated that group differences in memory increment from Trial 1 to Trial 3 was greater on the PALT compared to that on the AVLT. In order to provide a more direct description for this result, memory increment was by subtracting the score on Trial 1 from the score on Trial 3 (Figure 2). This result suggested that the rate of learning in aMCI decelerated more for associative memory than for item memory.

In order to further confirm this result, we compared the learning rate in PALT (Trial 3 - Trial 1) between aMCI patients and NC group with learning rate in AVLT (Trial 3 - Trial 1) as a covariate. The group difference remained significant, *F* (1, 77) = 9.87, *p* = .002, η^2^ _*p*_ = 0.11. However, when the learning rate in PALT was controlled as a covariate, the group difference in learning rate in AVLT was no longer significant, *F* (1, 77) = 1.57. The results suggested that the learning rate group differences were larger in associative memory test than in item memory test.

The correlations between memory span (Trial 1) and learning rate (Trial 3 - Trial 1) were not significant in both AVLT (*r* = −.13, *p* = .253) and PALT for all participants (*r* = .20, *p* = .071). In addition, in aMCI patients, the correlation between memory span (Trial 1) and learning rate (Trial 3 - Trial 1) was not significant either in both AVLT (*r* = −.02, *p* = .886) PALT (*r* = .16, *p* = .336).

### Intertrial acquisition and consolidation

#### AVLT

**Figure 3:**
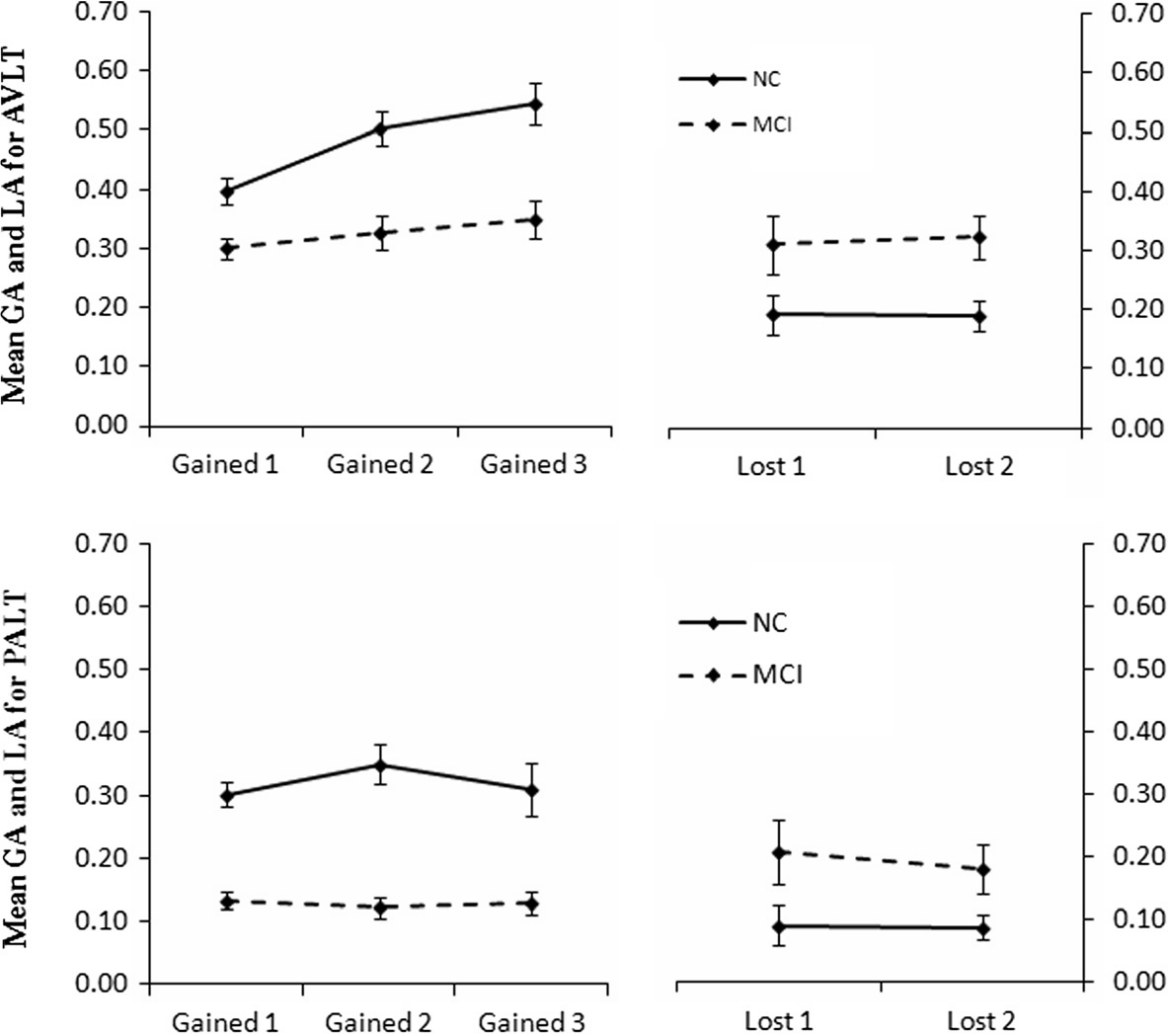
**GA and LA in the AVLT and PALT.** Values were calculated as proportions. Bars depict standard error of the means (SEM).

The means (across individuals) of GA and LA scores are shown in Figure 3. For GA, we found a significant main effect of group, *F* (1, 78) = 26.41, *p* < .001, η^2^ _*p*_ = 0.25, indicating that aMCI patients had obvious deficits in terms of acquisition. There was also a main effect of trial, *F* (2, 77) = 10.06, *p* < .001, η^2^ _*p*_ = 0.21, with an increase in scores on the latter two than the first learning trial. The interaction between group and trial was not significant, *F* (2, 77) = 2.74. For LA, we found a significant main effect of group, *F* (1, 78) = 8.31, *p* = .005, η^2^ _*p*_ = 0.10, with aMCI patients showing more rapid forgetting between trials. The effect of trial and the interaction between group and trial were not significant, both *F*s (1, 78) < 1.

#### PALT

We found that aMCI patients had obvious deficits in terms of GA, *F* (1, 78) = 57.67, *p* < .001, η^2^ _*p*_ = 0.43. However, there was no significant main effect of trial, *F* (2, 77) < 1, and no interaction between group and trial, *F* (2, 77) = 1.21. For LA, we found a significant main effect of group, *F* (1, 78) = 5.20, *p* = .035, η^2^ _*p*_ = 0.05, with aMCI patients showing more rapid forgetting between trials. There was no significant main effect of trial, *F* (1, 78) = 1.74, and no interaction between group and trial, *F* (1, 78) = 1.49.

Pearson correlations were performed separately for NC and aMCI between GA (the average GA score for three trials) and LA (the average LA score for two trials). The results indicated that GA and LA were uncorrelated in either group for both AVLT (NC, *r* = −.10; aMCI, *r* = −.06) and PALT (NC, *r* = .04; aMCI, *r* = .08), suggesting they were independent with each other.

### Discriminative power for detecting aMCI

**Figure 4:**
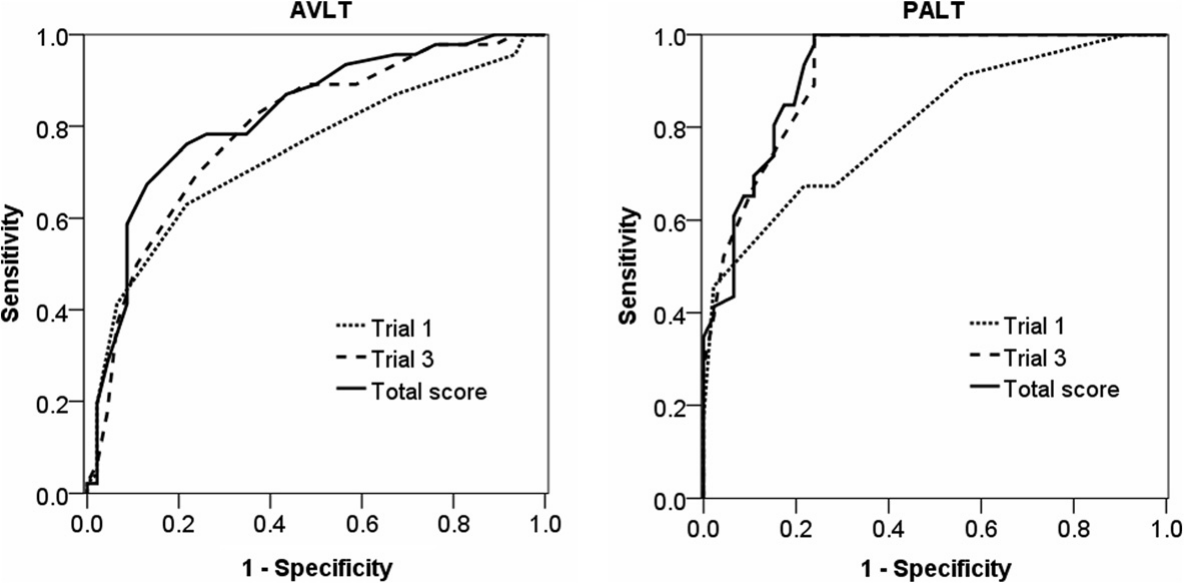
ROC curves plotting discriminative power of the PALT and AVLT.

In order to compare the discriminative power of the associative memory test and the item memory test with multiple trials for detecting aMCI from normal aging, the ROC curve analysis was used separately for the first trial, the third trial, and the aggregate total score on trials 1–3 in both tests (see Figure 4). For both the PALT and AVLT, the AUC was the highest for total score [for PALT, AUC = .923, 95% confidence interval (CI): 0.869–0.977; for AVLT, AUC = .824, CI: 0.738–0.911]. The AUC was higher for the third trial [for PALT, AUC = .920, CI: 0.866–0.974; for AVLT, AUC = .794, CI: 0.702–0.887] than for the first trial [for PALT, AUC = .805, CI: 0.718–0.893; for AVLT, AUC = .740, CI: 0.637–0.843], indicating that discriminative power increased when the number of trials increased. On the other hand, the AUC of the PALT was higher than AVLT in both the first trial (.805 vs. .740), the third trial (.920 vs. .794), and the total score (.923 vs. .824), which revealed that the associative memory test had more discriminative power than the item memory test in differentiating aMCI from normal aging. More importantly, the difference in AUC between PALT and AVLT was more obvious in the third trial (.920 - .794 = .126) than in the first trial (.805 - .740 = .065).

In order to confirm this result, we analyzed the percentage of instances where aMCI was accurately differentiated from normal aging by using a univariate logistic regression for each corresponding condition in both the PALT and AVLT (see Table 2). For both PALT and AVLT scores, accurate classification was the highest for total scores and was higher for Trial 3 than for Trial 1. Importantly, the differences in accurate detection between the PALT and AVLT were more obvious in Trial 3 (82.6% minus 72.8% is 9.8%) than in Trial 1 (72.8% minus 70.7% is 2.1%). This pattern was consistent with the results of ROC analysis.

**Table 2:** Accuracy of the PALT and AVLT in detecting aMCI by logistical regression analysis

		***B***	***SE***	**Wald**	**Accurate classification (%)**
AVLT	Trial 1	8.01	1.82	19.46***	70.7
	Trial 3	6.50	1.32	24.29***	72.8
	Total score	3.16	0.62	26.08***	77.2
PALT	Trial 1	15.27	2.83	29.01***	72.8
	Trial 3	12.65	2.33	29.38***	82.6
	Total score	5.61	1.04	29.04***	83.7

*Notes*. *** *p* < .001.

## Discussion

We examined differences in the rate of learning and intertrial performance on an item memory test in contrast with an associative memory test between people with aMCI and healthy controls and the ability of these tests to distinguish between aMCI and normal aging.

### Rate of learning

People with aMCI obtained significantly lower scores in terms of memory span (first trial) and rate of learning on both tests. These results are in line with those of previous studies [14,24]. As pointed out in previous studies [12,22], however, the interpretation of this deficit in learning rate needs to be conducted with caution. We cannot rule out the possibility that the interaction was due to scale effects [35,36] since there were clear differences in score in Trial 1 of both tests. In the AVLT, fortunately, although the performance of aMCI in Trial 2 was similar to the performance of NC in Trial 1, the learning curve of aMCI from trials 2–3 was still shallower than the curve of NC from trials 1–2, which provided evidence to some extent that people with aMCI learn slower than healthy controls.

The distinction between memory span and learning rate may be regarded as reflecting two different aspects of the learning process. For instance, subjective memory in multiple sclerosis patients is associated with initial-trial learning performance (i.e., memory span) but unrelated to recall performance on subsequent learning trials or aggregate learning scores [37], while patients with Asperger’s syndrome exhibited impairment only in the later trials but not in the initial trial of a multi-trial recall [38]. In the present study, the non-significant correlations between memory span and learning rate supports the notion that they reflect two different aspects of the learning process. In addition, although both memory span and learning rate are impaired in aMCI patients, there is no correlation between them in either test, indicating that memory span and learning rate are impaired independently.

Notably, in the current study, we found that people with aMCI had a slower learning rate for associative memory than for item memory, which provided additional evidence that associative memory was more impaired than item memory in aMCI, as what has been reported by investigation of memory span and total score in multiple learning trials. The results did not change even when the digit span forward, digit span backward, verbal fluency, and block design test were controlled as covariates, which suggested that the more severe impairment of learning rate in associative memory in aMCI patient is not because of their lower performance in executive functions.

Learning increment in multiple repeated trials reflect the ability of organizing information efficiently and of the development of higher-order memory units [8]. Subjective organization refers to a person’s ability to associate seemingly unrelated events in memory [39]. The ability of subjective organization is predictive of performance of multiple trial recall [40]. Therefore, the slower learning rate in aMCI patients indicates their impairment in organizing the repeated material into appropriate higher-order memory units, resulting in a failure to facilitate, or even in retardation, in retrieval processing. Furthermore, as revealed in this study, this organization of repeated materials seems to be even more impaired for item-item bindings than for separate items in aMCI patients. This is in line with the idea that the ability to bind items relies heavily on the hippocampus and entorhinal cortex [13], which are affected the earliest in aMCI [41-43]. Note, however, that aMCI patients’ performance on memory tests increased statistically with increasing trials; thus, the deficit in learning rate in aMCI patients is not as serious as that in AD patients [12].

### Intertrial acquisition and consolidation

Our finding that GA and LA were uncorrelated supports that they are relatively independent processes [12,22]. We separated the rate of learning into intertrial GA and LA in order to investigate the separate contributions of failure in acquisition and failure in consolidation to the shallower learning curve in aMCI patients. We found that aMCI patients exhibited significant damage in both acquisition and consolidation between adjacent trials in both the PALT and AVLT. The finding suggested that the substantial decline in GA and more rapid forgetting underlie aMCI’s shallower learning curve not only for item learning test as previously studies reported, but also for associative learning test. Previous studies have found that this deficit in intertrial acquisition and consolidation in aMCI patients is similar to that found in AD patients, although the extent of deficits was not as extreme compared to in AD [12,22]. Therefore, our results, together with those of previous studies, contribute to a deeper understanding of memory function impairment in aMCI and indicate areas that can be targeted for intervention.

### Discriminative power for aMCI

Both ROC curve and logistical regression analysis revealed that the third learning trial of both tests were able to differentiate aMCI from normal aging better than the first trials. Our results extend the results of previous studies, which found that the discriminative power of item list learning tests increases with increasing number of trials (>3 trials) [4,9], by showing the same pattern for item-item association list learning tests (even < 3 trial). Our results also correspond with Petersen et al.’s observation that the inability to acquire information in MCI becomes more apparent over several learning trials [44]. This phenomenon is related to the difference in learning rate between aMCI patients and healthy controls, which becomes larger as learning progresses, resulting in the higher discriminative power in the later trials than in the former trials. Not surprisingly, the total scores (i.e., across all trials) on both the AVLT and PALT were able to differentiate the two groups better than the scores of individual trials because the total score accumulated all the group differences in each trial.

We found that the discriminative power of the associative memory test was greater than for the item memory test in Trial 1, Trial 3, and total score, confirming the results of Troyer et al. (2008) [14] that associative memory test had advantage for identifying aMCI from normal aging in comparison to item memory. More importantly, beyond that, the present study provided evidence that the differences in discriminative power for aMCI between the PALT and AVLT tended to be larger in later trials than in earlier trials. The pattern was consistent when using both the ROC curve and the logistical regression analysis. Thus, taken together, these results support our hypothesis that associative memory tasks tended to be better for discriminating aMCI from normal aging with the number of trials increasing. Therefore, associative memory tasks with multiple learning-test trials may be particularly useful instruments for the detection of potential aMCI individuals.

The early detection of aMCI is very important because intervention might be most valuable at the earliest stages for those who are either recently diagnosed or at risk for developing it [45-47]. To aid the early detection of aMCI, clinicians and researchers are increasingly relying on neuropsychological assessment, genetic testing, and the use of neuroimaging [48]. However, genetic and neuroimaging methods are not appropriate for larger-scale screening in community-dwelling elderly because of their high cost and inconvenience. Therefore, for people with aMCI who are characterized by memory impairment, neuropsychological assessments with high discriminative power for screening or detecting memory decline at a very early stage would be more practical and helpful. However, the advantage of associative memory and multiple-trial learning tests suggested in previous studies [4,14] were seemingly ignored. The present study indicates further that an associative memory test with multiple learning-test trials is more powerful in amplifying the memory difference between aMCI and normal elderly. Therefore, an associative memory test with multiple learning-test trials would be a very useful instrument for the detection of potential “early aMCI,” a condition wherein elderly individuals’ memory performance is between the normal stage and conventional aMCI [45]. Further research is needed to confirm and develop this finding.

### Limitations

There are several limitations in the present study, especially regarding the assumption of the homogeneity of the PALT and AVLT that should be acknowledged. Firstly, the AVLT is a free recall test. In the PALT, however, participants were required to recall the second word in the pair only after the first word was given. Thus, the recall in the PALT resembles a cued recall rather than a free recall. On the other hand, there is some advantage to this “half-free” recall in the PALT. As performance on a free recall associative memory test is somewhat dependent on item scores (participants would be unable to report the associations if they could not recall the items) [14], the “half-free” recall in PALT reduces this effect by providing the first word in each associative pair. Secondly, the number of items or associations was not equal in these two tests. Previous studies have argued that the sensitivity of word list learning tests may be enhanced by increasing the number of items to be remembered (>10 items) [4,9]. The current study, however, revealed that the PALT, which has 12 items (association), had more discriminative power than the AVLT, which has 15 items, regardless of how the scores were analyzed. This finding provides further evidence for the idea that associative memory tests may be particularly sensitive to aMCI compared to item memory tests [14]. Thirdly, the presenting order of the two tests was not balanced, as the same CRF (Case Report Form) was applied to all participants. The PALT was always presented first, followed by a series of neuropsychological tests (all non-memory), and then the AVLT. But if the AVLT was influenced by the PALT due to proactive inhibition effect, then the impairments in PALT would have been underestimated. Even this, we still found more deficits in PALT than in AVLT. Therefore, we think the presentation order of the tests would not have led to negative effects for our findings.

Though all of the above factors differed between the two tests, they all favored PALT. Thus, they would not affect our findings of more impairment in PALT in principle. But we have to acknowledge the following two factors which may have confounded our findings. One concern is there were time restrictions on recall during the associative task that were not present in the item task; the other is that stimuli on the item task were presented in the same order on each trial, whereas they were presented in a different order on each trial of the associative task. As it would be easier to learn information over repeated trials when stimuli are presented in the same order each time, and easier to recall learned items when there are no time restrictions, these two factors may have confounded our conclusions to some extent.

In addition, although there is no significant difference in word frequency and age of acquisition between the two tests, Almond et al. (2013) [20] found that high-frequency words are learned at a higher rate than low-frequency words in older adults compared to younger adults, which indicates that the memory deficits may be modulated by word frequency. However, the number of words used in the present study is too limited to be separated into high and low frequency words for further statistical analysis. Future research on the learning rate of aMCI with a consideration of word frequency is needed.

Lastly, as mentioned in the Methods section, aMCI patients were not able to further classify into single- or multi-domains due to large sample based norms unavailable. Thus, we are unsure whether the results would differ between single- and multi-domain aMCI subtypes.

## Conclusions

Three main conclusions can be drawn from the present study. First, the deficit in learning rate in people with aMCI was more severe for associative memory than for item memory. Second, this deficit in associative memory was due to impairment in both intertrial acquisition and consolidation. Third, the advantage of aMCI-discriminative power in associative memory tests compared to item memory tests tended to be larger when the number of learning-test trials increased. Thus, associative memory tests with multiple learning trials may be particular useful instruments for the early detection of aMCI.

## Abbreviations

MCI: Mild cognitive impairment; aMCI: Amnestic mild cognitive impairment; AD: Alzheimer’s disease; NC: Normal control; AVLT: Auditory Verbal Learning Test; PALT: Paired Associative Learning Test; MMSE: Mini-mental state examination; GA: Gained access; LA: Lost access; SD: Standard deviation; ROC: Receiver operating characteristic; AUC: Areas under the curve.

## Competing interests

All authors declare that they have no conflicts of interest, including no financial, personal or other relationships with other people or organizations.

## Authors’ contributions

PYW collected the data, analyzed and interpreted data, drafted the manuscript. JL conceived the idea, designed the study, and participated in writing up and revising the manuscript. HJL assisted data analysis and paper writing. SZZ helped with clinical diagnosis and paper writing. All authors read and approved the final manuscript.

## Supplementary Material

Additional file 1 **The word frequency and age of acquisition in AVLT and PALT.***Note*. The numbers under the column “word frequency” refer to the number of the word in the corpus (20 million Chinese characters) of the “Dictionary of Usage Frequency of Modern Chinese Words” [49]. AoA = age of acquisition. AoA ratings were obtained following the procedure of Gilhooly and Logie (1980) [50]. A 7-point scale was used. The scale ranged from 1 (age 0–2) to 7 (age 13 and older). Intermediate points on the scale were identified with 2-year age bands. There were 45 adult (24 women and 21 men, aged 28.58 ± 4.25) participants. There was no significant difference between the two tests in word frequency (AVLT: M = 826.07, SD = 989.81; PALT: M = 845.17, SD = 1061.14, *t* = 0.05, *p* = .962), or AoA (AVLT: M = 2.52, SD = 0.50; PALT: M = 2.89, SD = 0.73, *t* = 1.53, *p* = .139).
Click here for file
