# Supplementary material for: Differences in learning rates for item and associative memories between amnestic mild cognitive impairment and healthy controls
Source: Behav Brain Funct. 2013 Jul 25;9:29. doi: 10.1186/1744-9081-9-29 (PMC3751153; doi:10.1186/1744-9081-9-29)
Supplement: Additional file 1 — The word frequency and age of acquisition in AVLT and PALT.Note. The numbers under the column “word frequency” refer to the number of the word in the corpus (20 million Chinese characters) of the “Dictionary of Usage Frequency of Modern Chinese Words” [49]. AoA = age of acquisition. AoA ratings were obtained following the procedure of Gilhooly and Logie (1980) [50]. A 7-point scale was used. The scale ranged from 1 (age 0–2) to 7 (age 13 and older). Intermediate points on the scale were identified with 2-year age bands. There were 45 adult (24 women and 21 men, aged 28.58 ± 4.25) participants. There was no significant difference between the two tests in word frequency (AVLT: M = 826.07, SD = 989.81; PALT: M = 845.17, SD = 1061.14, t = 0.05, p = .962), or AoA (AVLT: M = 2.52, SD = 0.50; PALT: M = 2.89, SD = 0.73, t = 1.53, p = .139). [file 1744-9081-9-29-S1.doc]

Additional files

### Additional file 1 – The word frequency and age of acquisition in AVLT and PALT

|  | To–be-remembered items (in Chinese) | To-be-remembered items (in English) | Word frequency | AoA |
| --- | --- | --- | --- | --- |
| AVLT | 斧子 | axe | 18 | 2.74 |
|  | 手臂 | arm | 24 | 2.20 |
|  | 闹钟 | alarm clock | 26 | 1.89 |
|  | 锤子 | hammer | 28 | 2.86 |
|  | 猫 | cat | 134 | 2.11 |
|  | 椅子 | chair | 137 | 1.97 |
|  | 耳朵 | ear | 246 | 3.83 |
|  | 自行车 | bike | 258 | 1.51 |
|  | 狗 | dog | 676 | 2.51 |
|  | 床 | bed | 981 | 2.89 |
|  | 刀 | knife | 1366 | 4.03 |
|  | 眼睛 | eye | 1488 | 2.69 |
|  | 汽车 | car | 1761 | 1.51 |
|  | 飞机 | plane | 1783 | 1.94 |
|  | 马 | horse | 3465 | 1.51 |
| PALT | 牛马 | oxen and horses | 72 | 3.09 |
|  | 粉红 | pink | 87 | 2.60 |
|  | 月亮 | moon | 123 | 2.29 |
|  | 电灯 | lamp | 155 | 3.26 |
|  | 躺下 | lie down | 413 | 2.46 |
|  | 东方 | east | 424 | 1.94 |
|  | 长短 | length | 438 | 3.51 |
|  | 服从 | submit | 531 | 1.51 |
|  | 衣服 | clothes | 625 | 1.54 |
|  | 铁路 | railway | 1429 | 1.77 |
|  | 玻璃 | glass | 2390 | 1.89 |
|  | 容易 | easy | 3455 | 2.49 |

*Note.* The numbers under the column “word frequency” refer to the number of the word in the corpus (20 million Chinese characters) of the “Dictionary of Usage Frequency of Modern Chinese Words” . AoA = age of acquisition. AoA ratings were obtained following the procedure of Gilhooly and Logie (1980) . A 7-point scale was used. The scale ranged from 1 (age 0–2) to 7 (age 13 and older). Intermediate points on the scale were identified with 2-year age bands. There were 45 adult (24 women and 21 men, aged 28.58 ± 4.25) participants. There was no significant difference between the two tests in word frequency (AVLT: M = 826.07, SD = 989.81; PALT: M = 845.17, SD = 1061.14, *t* = 0.05, *p* = .962), or AoA (AVLT: M = 2.41, SD = 0.78; PALT: M = 2.36, SD = 0.67, *t* = 0.182, *p* = .857).
